# Clinical significance of the overgrown follicles in dromedary camels: prevalence, risks, hemodynamics and response to prostaglandin F2α

**DOI:** 10.3389/fvets.2025.1723641

**Published:** 2025-12-10

**Authors:** Ahmed Ali, Derar R. Derar, Yousef M. Alharbi

**Affiliations:** 1 Department of Clinical Sciences, College of Veterinary Medicine, Qassim University, Buraydah, Saudi Arabia; 2 Department of Medical Biosciences, College of Veterinary Medicine, Qassim University, Buraydah, Saudi Arabia

**Keywords:** camelid reproduction, ovary, follicular pathology, prostaglandin, ultrasonography, blood, infertility

## Abstract

**Aim:**

This study investigated the prevalence, associated risk factors, hemodynamic characteristics, and response to prostaglandin F₂*α* (PG) of overgrown follicles (OVGF) in dromedary camels.

**Materials and methods:**

In Experiment 1, 338 females were examined for breeding soundness during the breeding season to determine the prevalence and risk factors of OVGFs (>2 cm in diameter). In Experiment 2, 45 females were categorized by follicular structure and scanned with spectral Doppler ultrasonography. In Experiment 3, 14 barren females with OVGFs were given PG treatment and monitored for hormonal changes and fertility outcomes.

**Results:**

OVGFs were found in 16.6% of the camels examined, with single (55.5%), double (35.7%), and triple (8.9%) follicles. The majority of affected ovaries contained growing or mature follicles (60.6%) or corpora lutea (3.7%), while 35.7% lacked any additional structures. OVGFs were linked to ovarian hydrobursitis (OVHB, 50%), clinical endometritis (CE, 35.7%), normal genitalia (12.5%), and pregnancy (1.8%). Significant risk factors were OVHB (OR = 27.5; *p* = 0.002) and CE (OR = 24.7; *p* = 0.004). Larger and trabeculated OVGFs showed increased systolic and diastolic velocities, a lower resistive index, and a different pulsatility index, indicating improved vascularization with follicular advancement. Complete regression followed by conception occurred in 2/14 animals, partial regression in 8/14 (with one conception), and no regression in 4/14, indicating a limited response to PG therapy. PG administration increased estradiol-17β levels but did not significantly impact progesterone or prolactin levels.

**Conclusion:**

In conclusion, overgrown follicles are relatively frequent in dromedary camels and are frequently associated with genital pathologies, particularly ovarian hydrobursitis and endometritis. Their variable vascularization and limited response to prostaglandin treatment suggest that OVGFs may be a persistent follicular condition with low therapeutic reversibility, which has implications for camel fertility management.

## 1 Introduction

Cystic structures are the most common ovarian abnormalities found in South American camelids (1), dromedaries (2–4), and Bactrian camels (5). Their role in infertility remains unclear (1).

Camel follicular growth has a distinctive turnover. In non-mating females, the dominant follicle causes atresia; however, in some females, the dominant follicle grows larger than typical follicles (overgrown follicle, OVGF, >2 cm in diameter) (1, 6–10). The reason for some females’ continued follicular growth is unknown. It could be due to abnormal or insufficient LH release, or follicular insensitivity caused by atypical or diminished LH receptors (11). Elevated FSH levels may promote continued follicular growth (12), whereas low serum zinc levels have been found in affected camels (13) and women with polycystic ovarian syndrome (14). Metabolic disturbances and oxidative stress have also been implicated in the development of OVGF in dromedary camels (4). Some studies indicate that OVGFs can regress spontaneously without intervention (7, 15, 16). El-Bahr et al. (17) and Ghoneim et al. (18) found that these follicles have low estradiol-17β levels and respond poorly to exogenous GnRH.

Color Doppler ultrasonography (CDU) is a noninvasive method for assessing the vascularity of the internal genitalia in large animals. It has been used to measure follicular blood flow, providing insights into follicular function and revealing physiological events that were previously undetectable with conventional B-mode imaging (15, 19–22).

Ultrasonography is a reliable tool for tracking follicular and corpus luteum dynamics, but follicular size alone does not elucidate the dominance phenomenon (23, 24). The dominant follicle’s response to prostaglandin treatment is alleged to imitate its functional status (23, 25).

The purpose of this study was to determine the prevalence and risks associated with OVGF in dromedary camels, characterize their vascular features using Doppler ultrasonography, and assess their responsiveness to PG treatment to determine whether OVGFs are a physiological variation or a pathological condition affecting fertility.

## 2 Materials and methods

Qassim University’s Animal Care and Welfare Committee approved the study (approval no. 12/46-47).

### 2.1 Experiment 1 (prevalence and risk factors)

At the clinic, 338 female dromedary camels’ reproductive tracts were examined for breeding soundness, specifically the presence of an overgrown follicle (OVGF, >2 cm). Breeding history data, including age (<5 y vs. 5–10 y vs. > 10 y), parity (Nulliparous vs. Multiparous), body condition score (thin [scores 1, 2] vs. moderate [scores 3, 4] vs. heavy [score 5], (26)), milk production (milking vs. dry), and housing system (closed system vs. open system), were recorded and analyzed for OVGF associated risks. Standard transrectal, transvaginal, and ultrasonographic examinations (Sonoscape X3V, Hamburg, Germany) were performed. The ovaries were examined for structural characteristics, the uterus for contents and echogenicity, and the vagina and cervix for patency and the presence of discharges. Reproductive disorders were considered in the risk factor analysis. No random selection was used, and the entire accessible population was included to determine the prevalence of OVGFs and associated risks.

Female camels were considered clinically normal if the ovaries showed no pathological structures, the uterus had normal contents and echogenicity, and no cervical or vaginal discharge or abnormalities were found. Reproductive disorders such as clinical endometritis (CE) and ovarian hydrobursitis (OVHB) were identified and analyzed as potential risk factors.

### 2.2 Experiment 2 (hemodynamic)

The study included 45 female dromedary camels ranging in age from 5 to 12 years. A duplex B-mode (gray scale) ultrasound scanner with a linear array transrectal transducer was used to assess ovarian structures. The maximum cross-sectional diameter of the follicles was measured, and females were classified as having growing or overgrown follicles (*n* = 11 and 34, respectively). Female camels were purposefully chosen based on ovarian ultrasonography findings to represent distinct follicular categories: 34 camels with various types of OVGFs and 11 with growing follicles.

**Figure 1:**
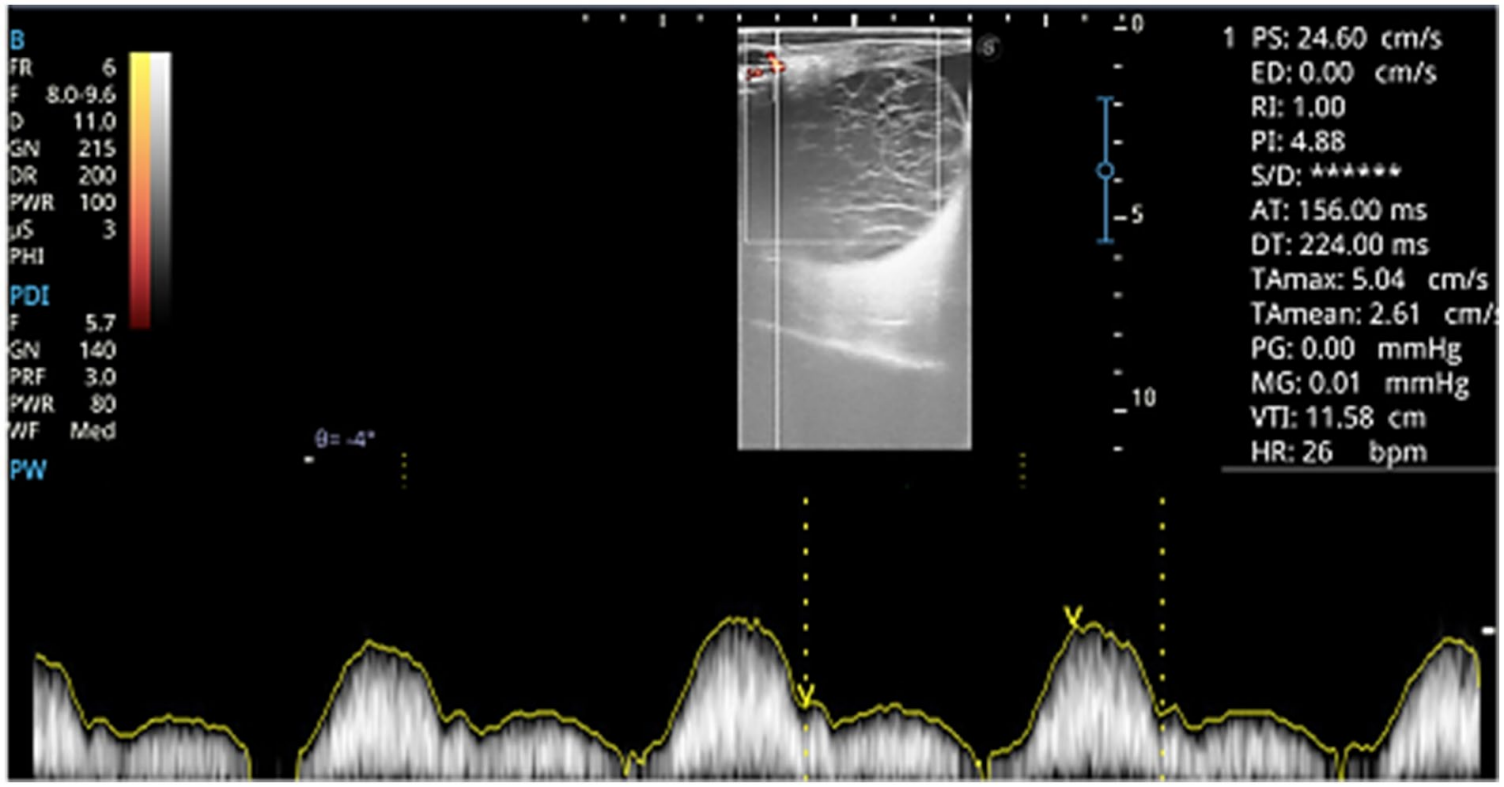
Spectral Doppler analysis of blood flow at the wall of an overgrown follicle (LF3). Parameters include peak systolic velocity (PSV), end-diastolic velocity (EDV), pulsatility index (PI), and resistive index (RI).

Spectral Doppler ultrasonography (PW mode) was used to measure blood flow in an arterial branch of the ovarian artery within the follicular wall (Figure 1). The ultrasound equipment was calibrated to the lowest detectable blood flow velocity (5 cm/s) using a probe frequency of 4.4 MHz in PW mode and 5.3 MHz in power mode, as well as a low gain setting to reduce imaging artifacts. Each follicle was assessed for the following hemodynamic parameters: peak systolic velocity (PSV), end-diastolic velocity (EDV), pulsatility index (PI), and resistive index (RI).

### 2.3 Experiment 3 (response to PG)

Fourteen barren female dromedary camels with overgrown follicles (OVGFs) were enrolled. Barren females were those who did not conceive during the current or previous breeding season despite being exposed to fertile males and had pregnancy ruled out by ultrasonography at the time of presentation. Female camels were included if they met a predefined criterion, which was the presence of an OVGF (>2 cm) on ultrasonography. This is purposive (criterion-based) sampling aimed at determining PG response in clinically relevant cases. Animals with systemic illness or unrelated reproductive pathology were disqualified. Breeding history and reproductive tract findings were documented.

The animals received three intramuscular injections of prostaglandin F₂*α* (500 μg cloprostenol sodium; Estrumate®, Vet Pharma, Germany), an intrauterine irrigation with 3% povidone-iodine solution (1,800 mL), and an intramuscular oxytetracycline (1 mL/10 kg, Terramycin LA, Zoetis®, Spain). Blood samples were taken from the jugular vein between 8:00 and 10:00 a.m. before and after treatment. The serum was centrifuged at 1,200 ×*g* for 10 min and stored for hormonal analysis. Competitive immunoluminometric assays (Shanghai International Hold Corp., Germany) were used to determine the concentrations of estradiol-17β (E2) and progesterone (P4). The intra- and inter-assay coefficients of variation were 6.11/8.17% for E2 and 4.37/7.12% for P4, with sensitivities of 8 pg./mL and 0.13 ng/mL, respectively. Prolactin (PRL) levels were determined with a camel-specific ELISA kit (Sunlong Biotech, China; CV 5.14/8.44%; sensitivity 0.1 ng/mL).

To clarify the relationship between the study components, Experiments 1–3 were carried out on the same clinical population but for different purposes. Experiment 1 analyzed all 338 females examined during the breeding season to determine prevalence and risk factors. Experiments 2 and 3 then used clearly defined subsets of this population, chosen based on ovarian ultrasonographic results. Experiment 2 included 45 females representing various follicular categories for hemodynamic assessment, while Experiment 3 included 14 barren females with OVGFs to assess PG response. These subsets were not designed to reassess prevalence, but rather to investigate mechanistic and therapeutic aspects, reducing confusion about overlap and selection bias. Als were classified as clinically normal according to a complete.

### 2.4 Statistics

Data are shown as mean ± SEM or percentages. The statistical analyses were carried out with SPSS software (version 25, IBM Corp., 2017). Multiple group comparisons were conducted using ANOVA, with pairwise comparisons using the least significant difference (LSD) test. The T-test is used to compare hormone levels prior to and following treatment. Binary logistic regression was used to investigate risk factors associated with the development of OVGF. The presence of OVGF was treated as a dependent variable, with age, parity, BCS, milk yield, CE findings, and ovarian hydrobursitis (OVHB) as independent variables. Pearson’s correlation coefficients were used to determine the relationship between follicle diameter and blood flow parameters. A *p* < 0.05 indicates statistical significance.

## 3 Results

### 3.1 Prevalence and risk factors

**Figure 2:**
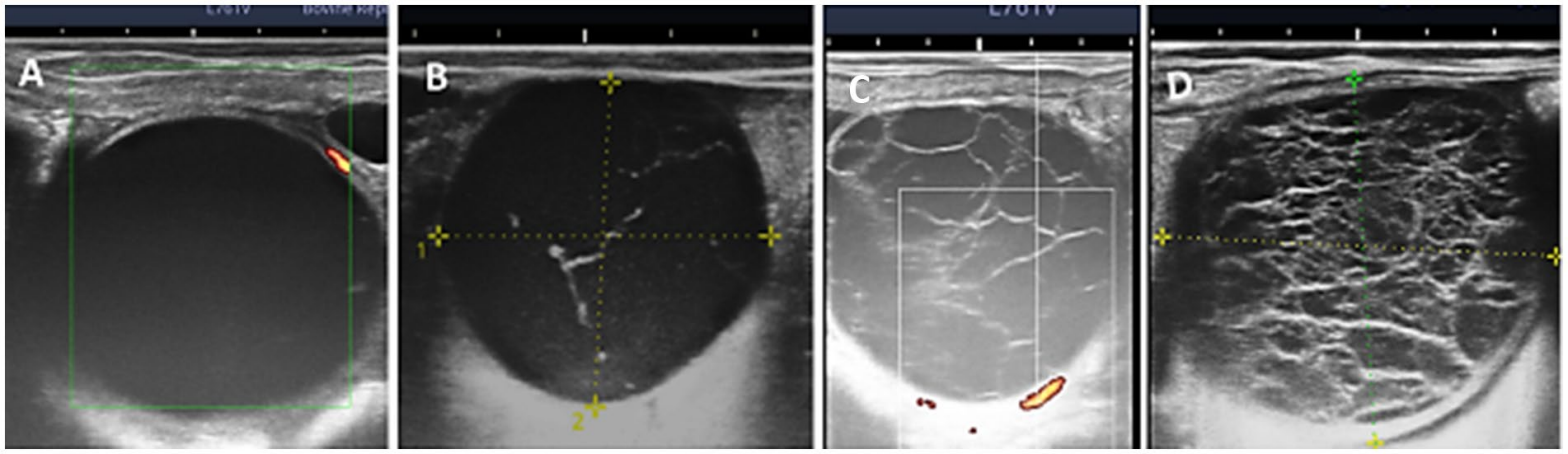
Ovaries of dromedary camels showing overgrown follicles with **(A)** clear content, **(B)** fine trabeculae, **(C)** moderate trabeculae, and **(D)** dense trabeculae. TH follicles had clear anechoic content and no trabeculae; LF1 had thin, sparse echogenic strands; LF2 had multiple, moderately thick strands occupying up to 50% of the lumen; and LF3 had numerous thick strands filling more than 50% of the lumen, resulting in a heterogeneous appearance.

OVGF was found in 56/338 (16.6%) female dromedary camels. Single (31/56, 55.5%), double (20/56, 35.7%), and triple (5/56, 8.9%) OVGFs were found in these cases. There were three distinct echo textures: (1) OVGFs with obvious hyperechogenic content (10/86, 11.6%); (2) OVGFs with few fibrous trabeculae (29/86, 33.7%); and (3) OVGFs with numerous echogenic transecting fibrinous strands (47/86, 54.7%) (Figure 2). The echo texture of camels with multiple OVGFs was similar in 42/55 (76.4%), but different in 13/55 (23.6%) cases. In the OVGF cases, 34/56 (60.6%) of the ovaries contained growing or mature follicles, 2/56 (3.7%) had corpora lutea at the same time, and 20/56 (35.7%) had no additional structures.

OVGFs were found in association with ovarian hydrobursitis (28/56, 50%), clinical endometritis (20/56, 35.7%), apparently normal genitalia (7/56, 12.5%), and during pregnancy (1/56, 1.8%). OVHB and CE were identified as risk factors for OVGF (Table 1).

**Table 1:** Logistic regression model for risk factors associated with the development of overgrown follicle in dromedary camels.

Variables	B	S.E.	Wald	df	Sig.	Exp (B)	95% C.I. for EXP (B)	
							Lower	Upper
Age	−0.020	0.513	0.002	1	0.969	0.980	0.358	2.681
Parity	0.530	0.895	0.350	1	0.554	1.699	0.294	9.816
Milk yield	0.040	1.584	0.001	1	0.980	1.041	0.047	23.234
BCS	−1.139	0.663	2.948	1	0.086	0.320	0.087	1.175
Housing	1.448	0.944	2.353	1	0.125	4.253	0.669	27.041
CE	3.207	1.118	8.227	1	0.004	24.702	2.761	221.029
OVHB	3.314	1.050	9.972	1	0.002	27.502	3.516	215.139
Constant	−5.976	2.524	5.607	1	0.018	0.003		

Hosmer–Lemeshow Chi^2^ = 5.723, df = 8, *p* = 0.678 (no evidence of poor fit). Cox and Snell *R*^2^ = 0.3794 (good logistic models often have pseudo *R*^2^ between 0.2 and 0.4). BCS, body condition score; CE, clinical endometritis; OVHB, ovarian hydrobursitis.

### 3.2 Hemodynamics characteristics

Table 2 comparing the hemodynamic properties of overgrown follicles (OVGF) in barren female camels with growing follicles. PSV levels rose significantly between the GF and LF1 subgroups. There was a positive correlation found between PSV and follicular diameter (*r* = 0.448, *p* = 0.001). The LF2 and LF3 groups had significantly higher EDV levels than the GF and TH groups (*p* = 0.04). PI values gradually increased from the GF to the TH subgroup. In contrast, RI gradually decreased from GF to LF3 groups, and there was a significant negative correlation between RI and follicular diameter (*r* = −0.326, *p* = 0.03).

**Table 2:** Hemodynamic characteristics of the OVGF in barren female camels compared with growing follicles.

Item	GF *n* = 11	OVGF				
		TH *n* = 8	LF1 *n* = 6	LF2 *n* = 7	LF3 *n* = 13	*p* value
Diameter (cm)	1.81 ± 0.16^b^	4.01 ± 0.35^c^	3.6 ± 0.23^cd^	3.17 ± 0.13^d^	4.96 ± 0.32^e^	0.0001
PSV (cm/s)	13.46 ± 01.5^b^	12.05 ± 3.17^ab^	22.92 ± 4.71^c^	17.75 ± 2.95^bc^	16.42 ± 1.92^bc^	0.001
EDV (cm/s)	2.34 ± 1.1^ac^	0.723 ± 0.72^a^	5.31 ± 2.44^ab^	6.98 ± 2.83^bc^	6.53 ± 1.72^b^	0.04
PI	1.56 ± 0.39^ac^	4.4 ± 0.96^bc^	3.62 ± 1.61^bcd^	1.1 ± 0.68^ad^	1.86 ± 0.59^ad^	0.03
RI	0.79 ± 0.09^a^	0.87 ± 0.13^a^	0.8 ± 0.24^a^	0.66 ± 0.11^a^	0.61 ± 0.1^a^	0.1

SF, small follicles, <3 mm; GF, growing follicles, 3–20 mm; TH, overgrown follicles with clear content; LF1, overgrown follicles with fine trabeculae; LF2, overgrown follicles with moderate trabeculae; LF3, overgrown follicles with dense trabeculae. ^a–e^Values within the same row bearing different superscript letters differ significantly.

### 3.3 Response to PG treatment

**Figure 3:**
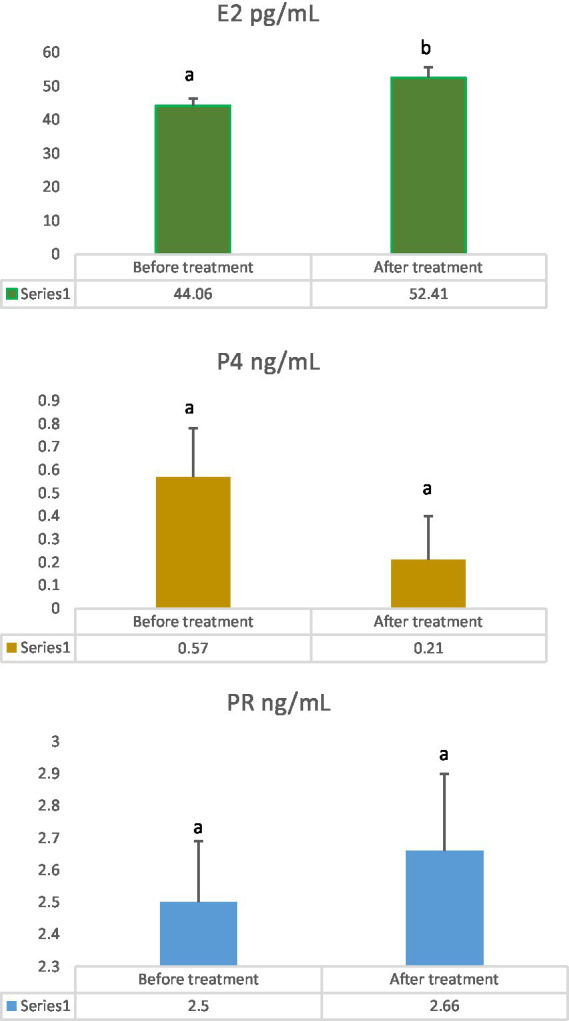
Effect of prostaglandin F₂α treatment on serum concentrations of estradiol-17β (E2), progesterone (P4), and prolactin (PR). Values in means ± SE, *n* = 14 (E2: 44.06 ± 2.24 vs. 52.41 ± 3.26; P4: 0.57 ± 0.21 vs. 0.21 ± 0.19; PRL: 2.5 ± 0.19 vs. 2.66 ± 0.24), before and after treatment, respectively. a, b: Mean significant difference, *p* < 0.05.

Table 3 summarizes the breeding history, clinical and hormonal profiles, and responses to PG treatment in female dromedary camels with OVGFs. Elevated serum P4 (>1 ng/mL) was found in three of fourteen camels, all of which had trabeculated OVGFs (Cam5, Cam7, Cam11). Two animals (Cam3, Cam5) exhibited estrus signs and conceived after mating, resulting in complete regression. Eight out of fourteen cases (Cam1, Cam4, Cam8, Cam9, Cam11, Cam12, Cam13, Cam14) showed partial regression or the development of a new OVGF, with four exhibiting estrus behavior and one achieving conception. There was no regression found in the remaining four camels (Cam2, Cam6, Cam7, Cam10). Hormonal analysis revealed a significant increase in mean serum E2 concentration (*p* = 0.04) following PG treatment, while mean P4 concentration decreased non-significantly (*p* = 0.1) and PR increased non-significantly (*p* = 0.6) (Figure 3).

**Table 3:** Breeding history, clinical and hormonal findings and response to PG-treatment in barren female dromedary camels with overgrown-follicles (*n* = 14).

Camel number	Before treatment							After treatment						
	Breeding history	Clinical findings	Ovarian structures		Hormones			Ovarian structures		Hormones			Estrus	Conception
			Right ovary	Left ovary	E2 (pg/mL)	P4 (ng/mL)	PR (ng/mL)	Right	Left	E2 (pg/mL)	P4 (ng/mL)	PR (ng/mL)		
Cam1	Anestrum = 2 m, Age = 12 y, Parity = 3, BCS = 4,	CE, Bilateral ovarian hydrobursitis	LF3 = 5.4 cm F = 1.7 cm	F = 1 cm	44.4	0.19	2.57	F = 2 cm, F = 1 cm, F = 0.5 cm	LF3 = 6 cm LF3 = 6 cm	36	0.17	3.14	No	–
Cam2	Anestrum = 3 m, Age = 8y, Parity = 5, BCS = 4	CE, ovarian hydrobursitis left side	F = 3 cm SF	F = 4.5 cm SF	43	0.15	2.86	F = 5 cm	F = 5.5 cm	45.4	0.16	2.79	No	–
Cam3	Repeat breeder (long-heat interval) = 9 m, Age = 6 y, Parity = 1, BCS = 3	App normal	F = 1.5 cm	LF1 = 3.1	40.4	0.18	2.1	F = 2 cm SF	F = 2 cm F = 2 cm	60.1	0.8	2.1	Yes	Yes
Cam4	Anestrum = 7 m, Age = 8 y, Parity = 1, BCS = 3	Apparently normal	LF2 = 6 cm	LF1 = 4 cm LF1 = 4 cm	41	0.2	3.43	LF1 = 5	F = 1.3	70.8	0.18	2	Yes	Yes
Cam5	Anestrum = 8 m, Age = 10 y, Parity = 4, BCS = 3	CE	LF1 = 5	SF	40	2.4	1.5	F = 2 cm F = 1.8 cm	SF	37.5	0.65	1.7	Yes	Yes
Cam6	Anestrum = 2 m, Age = 6 y, Parity = 1, BCS = 3	Narrow cervix	LF2 = 5 cm	LF2 = 2.5	40.8	0.15	3.6	LF = 4.5	F = 2	50.1	0.17	1.86	Yes	No
Came7	Anestrum = 6 m, Age = 8 y, Parity = 2, BCS = 5	CE	F = 2 cm F = 1.5 cm F = 1 cm	LF3 = 3 cm LF3 = 2.5 cm	45	1.9	1.7	F = 1 F = 2 cm	F = 4.5 cm F = 2 cm	60.2	0.15	1.6	Yes	Yes
Cam8	Anestrum = 6 m, Age = 9 y Parity = 2, BCS = 4	App normal	LF2 = 4.5 cm LF2 = 4.5 cm	SF	42.2	0.15	1.1	SF	LF2 = 4.5 F = 2	63.9	0.41	1.7	Yes	No
Cam9	Anestrum = 11 m Age = 12 y, Parity = 4, BCS = 5	App normal	LF1 = 3.5 cm	F = 4.5 cm	39.5	0.19	2.6	F = 5 cm	F = 1 cm F = 1.7 cm	52	0.15	4.43	Yes	No
Cam10	Anestrum = 11 m Age = 15 y, Parity = 6, BCS = 4	CE	static	LF3 = 4 cm	41	0.15	2.9	static	LF3 = 3 cm	66	0.2	2.71	Yes	Yes
Cam11	Anestrum = 7 m Age = 12 y, Parity = 5, BCS = 4	CE Bilateral ovarian hydrobursitis	LF2 = 12 cm	LF3 = 8.3 cm	72.4	1.9	3.2	F = 5 cm LF = 2 cm	F = 1 SF	67.8	0.3	2.8	Yes	No
Came12	Anestrum = 8 m, Age = 11 y, Parity = 4, BCS = 3	CE Salpingitis	LF3 = 6 cm	LF2 = 5 cm	39.2	0.15	2.7	LF3 = 6 cm	static	40	0.15	4.1	No	–
Cam13	Anestrum = 6 m, Age = 9 y, Parity = 2, BCS = 4	App norm	LF3 = 2 cm	LF3 = 6 cm	43	0.18	2.3	LF = 2 cm	static	41	0.16	3.1	No	–
Cam14	Anestrum = 3 m, Age = 8 y, Parity = 2, BCS = 3	App norm	LF3 = 3 cm	LF3 = 5 cm	45	0.15	2.5	LF = 2.1 cm	LF3 = 2	43	0.15	3.2	No	–

## 4 Discussion

The current study provides new information about the characteristics and clinical implications of OVGF in dromedary camels, focusing on prevalence, associated risks, hemodynamic features, and therapeutic response to PG.

The study approves the prevalence of OVGF in dromedary camels, loaning provision to previous findings that have divided view on whether this phenomenon is a normal physiological variation (7–9, 18) or related to reproductive disorders and endocrine dysfunction (2, 4, 11). A greater frequency of ovarian cysts was reported in infertile compared to fertile alpacas (1). Some females may be more liable to developing anovulatory follicles, this has been recognized to hormonal imbalances, metabolic disorders, and oxidative stress (4, 12, 27).

The risks associated with OVGF were CE and OVHB. There is strong scientific evidence that uterine diseases such as endometritis impair ovarian function in many domestic animals (28). Endometritis and purulent vaginal discharge in cattle are significant risk factors for abnormal progesterone profiles, prolonged luteal activity, and the formation of ovarian cysts (29, 30). Uterine inflammation also inhibits the LH surge, preventing ovulation and promoting cyst formation (31). Inflamed uterine cytokines such as IL-1β, TNF-*α*, and prostaglandins can directly affect ovarian tissue, reducing steroidogenesis and follicle viability (32). OVGF formation in camels may also result from endocrine and local ovarian dysregulation similar to that reported in other large mammals. Altered LH-receptor responsiveness, disturbed LH-surge patterns, and prolonged or dysregulated FSH activity, together with oxidative-stress–induced impairment of granulosa-cell function, have all been implicated in the development of persistent or cystic follicles (12, 33–35). Other endogenous and exogenous factors, such as photoperiod, male presence in the herd, and nutritional status, have been proposed to influence OVGF formation in camels (16). Furthermore, in Morocco, the prevalence of cystic follicles in female dromedary camels varies seasonally, peaking in April–May. However, similar to our findings, age and body condition had no effect on their occurrence (36), implying a limited effect of these factors under local management conditions.

Spectral Doppler ultrasonography improved understanding of follicular dynamics by quantifying blood flow characteristics. The progressive increase in PSV and EDV from GF to advanced OVGF categories (LF1-LF3) suggests better vascularization in larger, more structurally complex follicles. The decrease in RI and shift in PI indicate decreased vascular resistance and increased perfusion, both of which are commonly associated with follicular dominance and persistence (21, 37). These vascular patterns suggest that OVGF are well-perfused structures, which could prevent spontaneous regression in the absence of normal ovulatory function (15, 21, 37–39). While correlations between Doppler indices and follicular diameter suggest biologically relevant trends, such as improved vascularization and reduced vascular resistance in larger OVGF, we emphasize that these findings support, but do not definitively establish, the proposed physiological interpretation. The cited references (15, 21, 37–39) have been utilized to contextualize these trends within previously documented vascular changes in dominant and persistent follicles.

PG treatment was generally ineffective in resolving OVGF, with only two of fourteen camels achieving complete regression and conception. The low success rate suggests that OVGFs are extremely resistant to luteolytic intervention. This may also be mildly influenced by the small treatment sample, although its impact is likely limited. Anovulatory follicles in other domestic species are resistant to PG-induced regression (40, 41). These follicles may be hormonally unresponsive or functionally compromised. The lack of endocrine changes after PG treatment indicates that these follicles are hormonally inactive or functionally impaired (15, 17, 18). The prolactin is unlikely to have any role in OVGF persistence. As in our protocol, Zaher et al. (42) found that multiple doses of cloprostenol were needed to induce complete luteolysis and restore luteal function in dromedaries.

Camels’ luteinized follicles appear to differ from those of cattle in terms of P4 production and prostaglandin response (43, 44). Furthermore, trabecular density does not reliably predict progesterone secretion capacity. Only a few camels in this study responded to PG. Manjunatha et al. (7) discovered that plasma P4 concentrations were <1.0 ng/mL in 85% of follicular waves and >1.0 ng/mL in 15% of waves in the absence of spontaneous ovulation, which is consistent with and supports our findings. In the current study three camels with OVGF produced P4 > 1 ng/mL. Unexpectedly, an embryo recipient with a luteinized anovulatory follicle was able to sustain pregnancy and deliver a live calf in dromedary camels (45).

Unlike in cattle, milk production and the postpartum period appear to have little influence on the formation of cystic structures in camels, as all females tested were non-lactating and had previously given birth. Furthermore, in the current study, age, parity, body condition score (BCS), and housing system had no effect on the incidence of OVGF. Similarly, in Morocco, age and body condition had no significant effect on cyst frequency in dromedary camels (36).

Overall, the study found that OVGF are not only structurally abnormal, but also functionally robust due to increased vascularization. Improved blood supply promotes follicular growth and prevents luteolysis, rendering traditional PGF₂α therapy less effective. The ongoing presence of OVGF thus poses a significant barrier to fertility in dromedary camels, particularly when combined with concurrent uterine and ovarian disorders.

These findings are directly relevant to camel reproductive management in the Gulf region, where early detection of ovarian abnormalities is critical for reducing infertility losses. PGF₂α alone has limited efficacy in treating OVGF-related infertility. Concurrent uterine infections may require alternative or combined therapeutic strategies, such as hormonal synchronization protocols or ultrasound-guided follicular aspiration (2, 46). Second, Doppler ultrasonography emerges as a valuable diagnostic tool capable of not only detecting OVGF but also predicting its persistence based on vascularization patterns. This allows veterinarians to tailor reproductive management plans while avoiding ineffective treatments. Finally, the strong association between OVGF and other reproductive pathologies suggests that OVGF could be a marker of underlying reproductive dysfunction, necessitating a more comprehensive diagnostic approach.

This study has some limitations that should be considered. The PG-treated subgroup was relatively small, follow-up fertility data did not extend beyond initial conception, and histopathological confirmation of follicular structure was not available. In addition, hormonal profiling across estrous cycles was limited, which may restrict deeper interpretation of the endocrine mechanisms involved.

## 5 Conclusion

OVGFs are a common finding in dromedary camels and are frequently associated with reproductive health problems. Their poor response to prostaglandin treatment, as well as progressive vascularization, support the notion that they are a pathological condition rather than a physiological variation, with potentially negative fertility consequences.

## Data Availability

The original contributions presented in the study are included in the article/supplementary material, further inquiries can be directed to the corresponding author.
